# *Pseudomonas* sp. F204 Promoted Tomato Growth and Altered Rhizosphere Bacteria Community

**DOI:** 10.1007/s00284-025-04278-y

**Published:** 2025-07-12

**Authors:** Jiawei Li, Yingjie A., Minghao Liu, Xin Li, Yilin Zhan, Muhammad Khashi u Rahman, Xingang Zhou

**Affiliations:** 1 Key Laboratory of Biology and Genetic Improvement of Horticultural Crops (Northeast Region, Ministry of Agriculture and Rural Affairs), Heilongjiang Key Laboratory of Cold Area Protected Horticulture, Department of Horticulture, Northeast Agricultural University, Harbin, 150030 China; 2 School for the Engineering of Matter, Transport and Energy, Arizona State University, Tempe, AZ 85287 USA; 3 College of Agriculture, Northeast Agricultural University, Harbin, 150030 China; 4 Department of Microbiology and Genetics & Institute for Agribiotechnology Research (CIALE), University of Salamanca, 37007 Salamanca, Spain

## Abstract

Extracellular DNA (eDNA), as a potential plant autotoxin, can cause soil sickness, negatively affecting plant growth and reducing crop yield. Some microbial taxa can degrade eDNA; however, the effect of these bacteria on plant growth and rhizosphere microbial communities is unknown. In this study, eDNA-degrading bacterial strains were screened from soils, and their eDNA-degrading ability and plant-growth-promoting characteristics were tested in vitro. The effects of the most potent eDNA-degrading strain on tomato plant growth were assessed, and changes in the rhizosphere bacterial community were analyzed using high-throughput amplicon sequencing. Among a total of more than 600 isolated strains, *Pseudomonas* sp. F204 showed the strongest eDNA-degradation capacity and exhibited phosphate solubilization, siderophore, and indole-3-acetic acid production abilities. Pot experiment showed that inoculation of *Pseudomonas* sp. F204 significantly altered tomato rhizosphere bacterial community structure and composition, and stimulated the growth of tomato seedlings. Overall, this study shows that eDNA-degrading bacteria can alter rhizosphere bacterial community and promote plant growth, and highlights the important role of soil microbial community in alleviating autotoxicity in continuous cropping systems.

**Supplementary Information:**

The online version contains supplementary material available at 10.1007/s00284-025-04278-y.

## Introduction

Autotoxicity refers to the phenomenon whereby plants release certain compounds (i.e., autotoxins) that inhibit the growth of conspecific or closely related plants [1]. As a significant factor, it impedes soil sickness and poses a challenge to sustainable agricultural development. Autotoxins can be released into the soil environment through volatilization, leaching, root exudation, and residue decomposition, affecting the plant itself, and the same or heterogeneous plants growing on the same soil as soil properties change [2, 3]. In recent studies, extracellular DNA (eDNA) from plants such as tomato [4], cucumber [5], and faba bean [6] has been shown to act as an autotoxin, inhibiting the growth of conspecific seedlings.

DNA, a vital biomolecule present in every living cell, has also been found to contribute to autotoxicity in plants [4, 7]. Plant DNA can actively or passively be shed from cells by extrusion mechanisms or cell lysis, resulting in the release of eDNA [8]. Upon interaction with soil components such as minerals and humic substances, eDNA becomes fragmented rather than intact genomic DNA [9]. These eDNA fragments can accumulate in soil and act as autotoxic substances to plants. High concentrations of eDNA can inhibit seed germination, leading to seedling mortality and stunting plant growth, and posing a challenge to sustainable agricultural development [10].

Microorganisms are widely used to degrade organic and inorganic pollutants and promote plant growth, making them an environmentally friendly and safe option [11]. Members of numerous bacterial taxa, including *Bacillus*, *Pseudomonas*, *Microbacterium*, and *Sphingomonas*, have been shown to possess eDNA-degradation capabilities [12]. For example, zone of DNA hydrolysis was observed when *Bacillus* was applied to TBO-DNA agar plates [13]. Although certain plant-associated bacterial taxa have been shown to assist hosts under various biotic and abiotic stresses, their role in degrading eDNA to support the host remains unknown.

In addition, several bacteria possess plant-growth-promoting abilities through the siderophore and indole acetic acid production or solubilization of plant-unavailable forms of nutrients [14]. These plant-growth-promoting rhizobacteria (PGPR) can directly benefit the host plant or indirectly through the increase in the available nutrients in the soil and improve the nutrient use efficiency; all this can counteract the autotoxins-induced autotoxicity [15]. However, the ability of PGPR to promote plant growth depends on their successful interaction with native microbial communities, which play a crucial role in shaping host health [16]. For example, modulation of bacterial community composition by application of a PGPR *Bacillus shortum* was the main mechanism of maize plant growth promotion and yield increment [17]. Similarly, the application of *Pseudomonas* inoculants augments the abundance of several rhizosphere microbial taxa and significantly affects the microbial community structure in the rhizosphere soil [18]. These positive effects of PGPR-induced changes in microbial community composition on host plants are mainly due to the altered microbial functionality, thereby causing feedback effects on plant growth [19]. Therefore, it is important to investigate the previously unknown aspects of how eDNA-degrading bacteria affect the plant rhizosphere microbial community and plant growth.

Previous studies have shown that tomato eDNA has an inhibitory effect on tomato growth [4]. Multiple bacterial strains have been found to degrade eDNA [20, 21], but the effect of these strains on plant growth and rhizosphere bacterial community remains unknown. We hypothesized that tomato bacteria not only degrade eDNA, but also promote tomato growth and induce significant changes in the rhizosphere bacterial community. To test the hypothesis, we (1) screened *Pseudomonas* sp. F204 as a strong tomato eDNA-degrading bacteria using TBO-DNA agar medium; (2) evaluated its plant-growth-promoting abilities in vitro; and (3) tested its effects on tomato seedlings growth and rhizosphere bacterial community in a greenhouse pot experiment.

## Materials and Methods

### Experimental Soil

Soil sample (0–15 cm depth) was collected from the Horticultural Experimental Station of Northeast Agricultural University, Harbin, China (45° 41′ N, longitude 126° 37′ E). The soil was homogenized and sieved (2 mm) to remove stones and plant debris. This soil is classified as sandy loam and has the following characteristics: organic matter content of 46.05 g kg^−1^, inorganic nitrogen content of 66.37 mg kg^−1^, available phosphorus content of 56.56 mg kg^−1^, available potassium content of 125.78 mg kg^−1^, electrical conductivity EC (1:2.5, w/v) of 0.31 mS cm^−1^, and pH (1:2.5, w/v) of 7.43.

### Extraction and Preparation of Plant eDNA

To prepare plant tissues for DNA extraction, plant leaves were collected from 4-week-old tomato seedlings (Cv. DN708). Plant genomic DNA was extracted using a high-salt cetyltrimethylammonium bromide (CTAB) extraction protocol [22]. The quality and yield of extracted DNA were checked by 2% (w/v) agarose gel electrophoresis and NanoDrop 2000 spectrophotometer. Fragmented DNA was obtained by sonication using a SCIENTZ-950E ultrasonic processor. The fragment of DNA was verified by 2% (w/v) agarose gel electrophoresis [4].

### Soil Treatment with Plant eDNA

Ten grams of soil was transferred into 50 mL centrifuge tubes, and soil moisture content was adjusted to 40% of the soil capacity. Subsequently, the soil samples were incubated in the dark at 28 °C for 7 days. After this initial period, the soil moisture content was adjusted to 60% of soil capacity by adding water-soluble eDNA. At 0, 2, 4, 6, and 8 days after moisture adjustment, tomato-fragmented DNA was added to the centrifuge tubes to achieve a final concentration of 10 μg·g^−1^ of soil. Control tubes received an equal volume of sterile water. The soil samples were then further incubated under the same conditions for 10 days. Finally, soil samples were removed for bacterial isolation using the dilution plate spread method [23].

### Isolation and Screening of Bacteria

The dilution series was inoculated separately on the following media: (1) 1/10 strength tryptic soy agar (TSA; Difco), (2) nutrient agar, (3) R_2_A agar (Difco), (4) King's medium B agar, and (5) peptone yeast agar. All media were supplemented with 200 mg·L^−1^ Delvocid (DSM; active ingredient: natamycin) to prevent fungal growth, placed at 25 °C, and isolated after 3 days [24, 25]. We obtained a total of more than 600 strains of bacteria.

To determine whether the bacterial strains could degrade tomato DNA, validation was performed using TBO-DNA agar medium [26]. The medium was poured into Petri dishes to achieve a thickness of approximately 3 mm. After the agar solidified, wells were made, and 20 μL of bacterial supernatant was added to each well and incubated at 37 °C for 6–12 h [13]. Positive detection transparent zones confirmed the strain's ability to degrade DNA [27]. We measured the degradation ability of the obtained isolates and found that three isolates could degrade tomato eDNA, and we selected isolate F204 due to its strongest degradation effect for further experiments.

The bacterial genomic DNA was extracted from cells grown in LB medium using the Bacteria Genomic DNA Extraction Kit (Tiangen Corporation Ltd, Beijing, China) according to the manufacturer’s instructions. The 16S rRNA gene was amplified by PCR with the universal bacterial primers 27F and 1492R under conditions described previously [28]. The PCR products were subjected to 1.0% agarose gel electrophoresis and sent to Shanghai Sheng Gong Bioengineering Co. for 16S rDNA sequencing. The sequences measured were compared in the BLAST in NCBI (www.ncbi.nlm.nih.gov) to obtain homologous gene sequences, and a phylogenetic tree was constructed using the neighbor-joining algorithm in MEGA 11.0 software.

### Determination of Plant-Growth-Promoting Characteristics of Isolate

We further tested the plant-growth-promoting characteristics of the degrading strain F204 to determine the direct mechanisms underlying its role in plant growth promotion. The production of siderophores was assessed using Chrome Azurol S (CAS) agar plates [29] and quantified at 630 nm by Murakami's method [30]. The phosphorus solubilization activity of the strain was determined using the National Botanical Research Institute’s Phosphate (NBRIP) agar medium [31]. Quantification of phosphate was determined at 600 nm using Liu's method [32]. The indole acetic acid (IAA) production was assessed using the method outlined by Patten and Glick [33]. Absorbance at 530 nm was measured, and IAA concentration was estimated from a standard curve.

### Greenhouse Pot Experiment

The effect of bacterial isolate F204 on tomato plant growth and rhizosphere bacterial communities was evaluated by inoculating the strain into the soil. Tomato seeds were germinated at 26 °C and healthy tomato seedlings were planted in cropland soil. At the two true leave stages, seedlings were transferred to the pots (10 × 10 cm), each containing 500 g of soil. The tomato seedlings were grown in a greenhouse under controlled conditions (day temperature: 32 °C, night temperature: 22 °C; relative humidity: 60–80%; 16 h of light/8 h of darkness). The soil moisture content was maintained at approximately 65% of its water holding capacity.

Overnight cultures of the isolated strains in LB broth were centrifuged (5000 g, 5 min) and resuspended in phosphate-buffered saline to obtain a bacterial suspension. There were two treatments in the tomato pot experiment: (1) 10 mL of phosphate-buffered saline solution without bacteria (control), and (2) 10 mL of bacterial suspension. Treatments were applied to each pot as soil irrigation. Each treatment was replicated five times, with five pots per replicate, resulting in a total of 25 pots per treatment.

After 3 weeks of inoculation, tomato plants were harvested. The entire plant height was then measured using a straight-edge ruler, and the plant was weighed using an electronic balance. Subsequently, the plants were dried at 60 °C to constant weight to measure the total dry weight. Soil tightly adhered to the roots was removed by brushing with a sterile brush and considered as rhizosphere soil [34]. Five rhizosphere soil samples were collected per replicate and combined to create one composite sample for each replicate. The sampled soil was then sieved through a 2 mm mesh and stored at − 80 °C for DNA extraction.

### DNA Extraction and Illumina Miseq Sequencing

Genomic DNA was extracted from 0.25 mg of tomato rhizosphere soil using the Power Soil® DNA Isolation Kit (MO BIO Laboratories, CA, USA). DNA was extracted three times from each composite soil sample, and the extracted DNA solutions were pooled. The quality of the extracted DNA was checked by 1.2% (w/v) agarose gel electrophoresis.

Changes in tomato rhizosphere bacterial community composition were studied through high-throughput sequencing at Majorbio BioPharm Technology Co, Ltd (Shanghai, China). Briefly, the V3–V4 hypervariable region of bacterial 16S rDNA was amplified by PCR using F338 (5′- ACTCCTACGGGGGGCAG-3′)/806R (5′- GACTACHVGGGTWTCTAAT-3′) primers set [35], and the products were purified for Illumina Miseq sequencing. The purified amplicons were paired-end sequenced on an illumine MiSeq platform PE300 (Illumina San Diego, USA) according to the company’s standard protocol.

Raw sequences were processed using QIIME [36]. Trimming, alignment, quality filtering, culling of reads smaller than 200 base pairs, and removal of chimeras were performed [37]. Sequences were clustered into OTUs at 97% nucleotide identity using UPARSE [37]. Each OTU was taxonomically classified with the help of the SILVA database (v132). To avoid potential bias due to differences in sequencing depth, the number of sequences in each sample was reduced to 42,776.

### Statistical Analyses

Statistical analyses were conducted using R (v4.1.0, http://www.r-project.org/). Bacterial alpha diversity was analyzed using “vegan” and “picante” packages [38]. The principal coordinates analysis (PCoA) based on Bray–Curtis dissimilarity was used to explain the differences in bacterial community structure, and the permutation multivariate analysis of variance (PERMANOVA) was performed by “vegan” and “ggalt” package. The “edger” and “ggplot2” packages were used to visualize the distribution of differential OTUs at the bacterial phylum level. Welch's *t*-test was used for comparisons between the two groups, and statistical differences between groups were considered significant at *P* < 0.05.

## Results

### Isolation, Screening, and Identification of eDNA-Degrading Bacteria

**Fig. 1:**
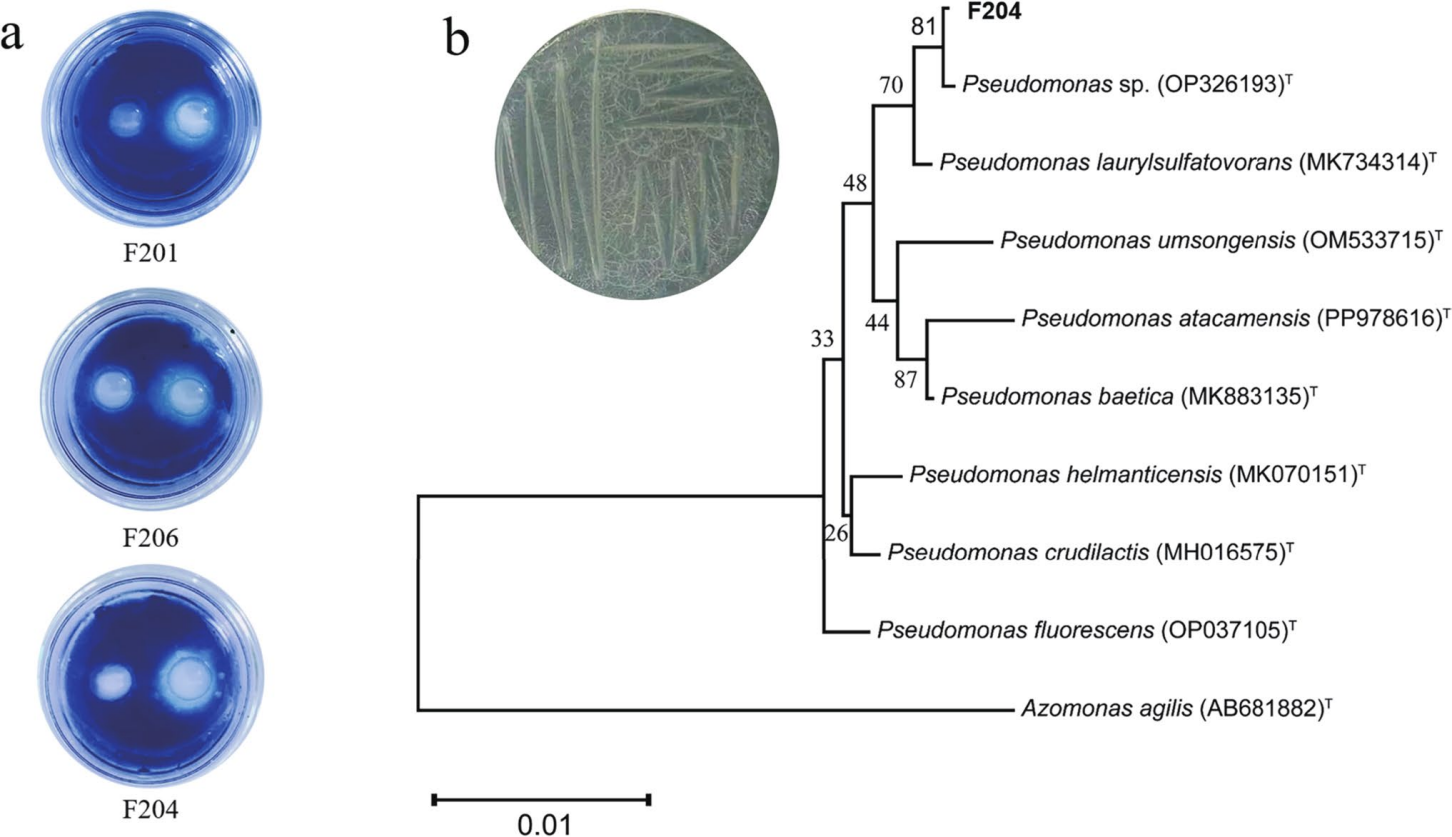
Screening and identification of isolates. **a** Assessment of bacterial eDNA-degradation ability, with white transparent circles indicating the degradation efficacy. **b** The neighbor-joining phylogenetic tree showing the evolutionary relationship of isolate F204, with the inset image showing the morphology of isolate F204. Bootstrap values are based on 1000 resamples and are shown at the branch points

More than 600 isolates were obtained from soil treated with tomato eDNA, but only three isolates showed color reactions on agar plates, with isolate F204 showing the strongest color reaction (Fig. 1a). Therefore, we selected isolate F204 for further experimentation. The taxonomic status of the isolate F204 was determined by constructing a phylogenetic tree based on the 16S rDNA sequences (Fig. 1b). At the genus level, the isolate F204 belonged to the *Pseudomonas* genus and was designated as *Pseudomonas* sp. F204. The sequence of isolate F204 has been uploaded to NCBI with accession number PQ638548.

### Assessment of Plant-Growth-Promoting Characteristics of the *Pseudomonas* sp. F204

**Fig. 2:**
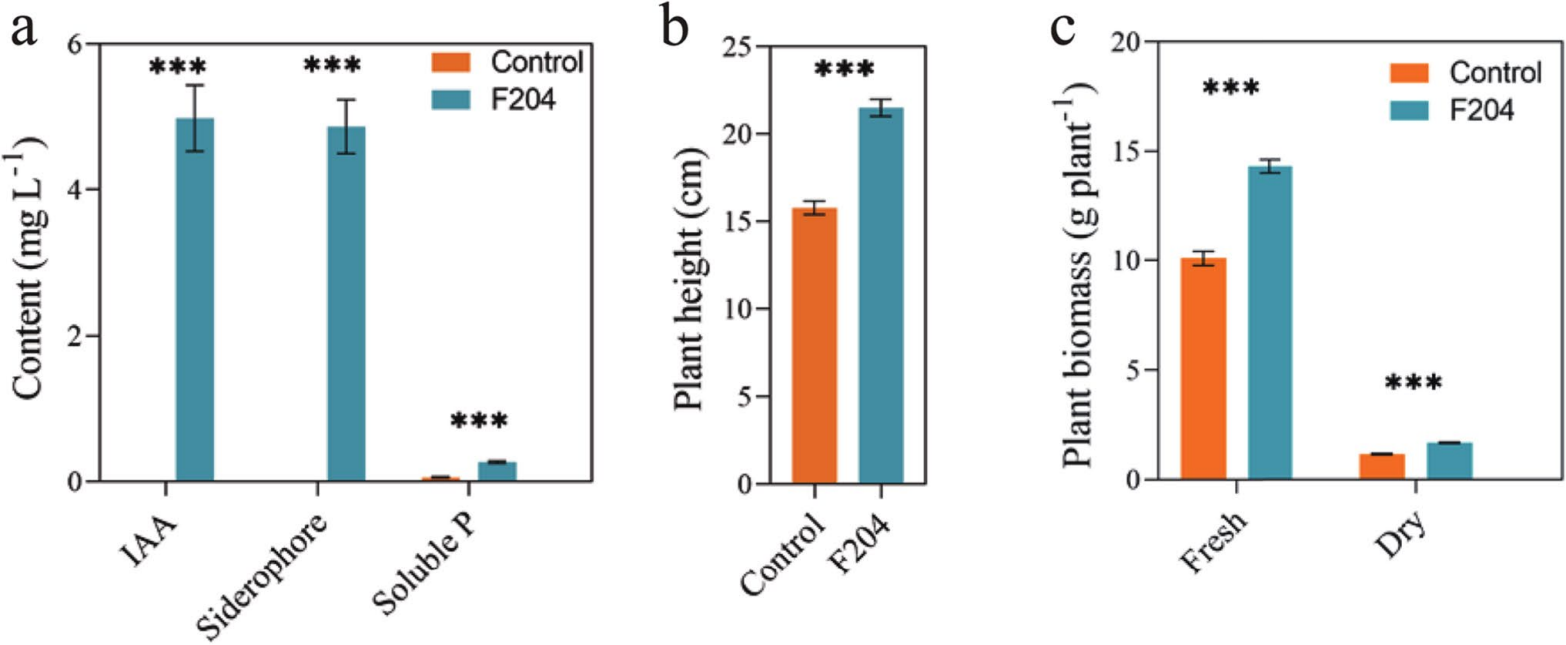
**a** Determination of plant growth promotion-related characteristics of *Pseudomonas* sp*.* F204, including IAA production assay, Siderophore production assay and Phosphate solubilization assay. **b** and **c** The effect of *Pseudomonas* sp. F204 on the growth of tomato seedlings. The asterisks show statistically significant differences between treatments (Welch's *t*-test, ***P < 0.001)

The results of in vitro experiments showed that *Pseudomonas* sp. F204 demonstrated the ability of phosphate solubilization, siderophore production, and IAA production (Figs. 2b, S1, S2; Welch's *t*-test, *P* < 0.05). In the pot experiment, compared to the control group, inoculation with F204 bacterial suspension significantly increased the height of tomato seedlings by 36.26% (Figs. 2b, S3; Welch's *t*-test, *P* < 0.05), fresh biomass by 41.87%, and dry biomass by 43.82% (Fig. 2c; Welch's *t*-test, *P* < 0.05).

### Effect of *Pseudomonas* sp. F204 Inoculation on Alpha and Beta Diversity of the Tomato Rhizosphere Bacterial Community

**Fig. 3:**
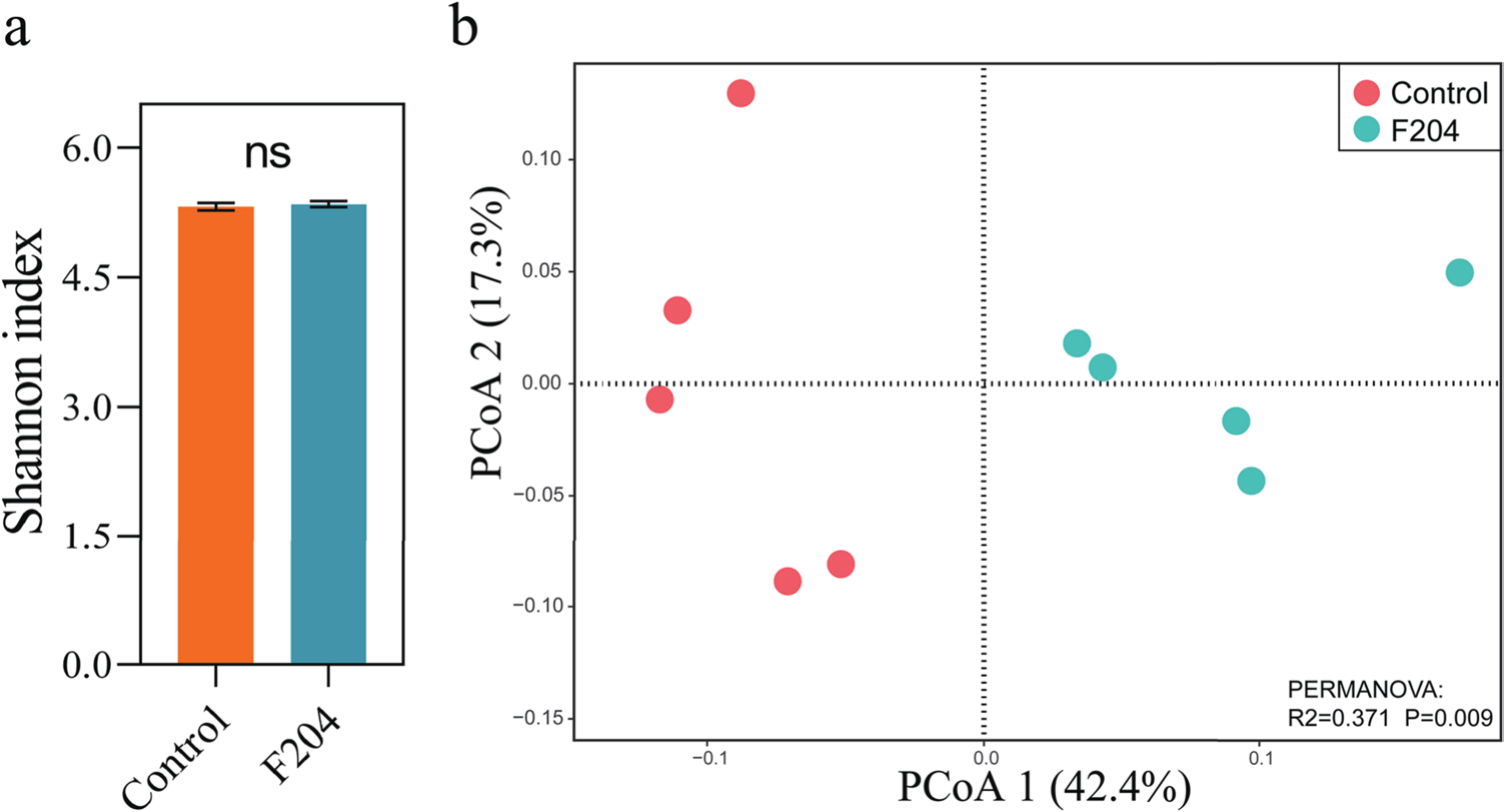
Changes in tomato rhizosphere bacterial alpha (**a**) and beta (**b**) diversity in response to *Pseudomonas* sp. F204 inoculation. ns: non-significant difference based on Welch's *t*-test, *P* < 0.05

A total of 659,328 reads were obtained by 16S rRNA gene amplicon sequencing of tomato rhizosphere soil samples using the Illumina platform. The tomato soil inoculation with *Pseudomonas* sp. F204 did not affect the alpha diversity (i.e., Shannon index) of the rhizosphere bacterial community (Fig. 3a). For bacterial beta diversity, PCoA based on the Bray–Curtis dissimilarity matrix showed a clear separation of *Pseudomonas* sp. F204-inoculated samples with a control. Briefly, the first PCoA1 contributed to 42.4% of the variation in bacterial community composition, while PCoA2 contributed to 17.3%. Together, PCoA1 and PCoA2 explained 59.7% of the total variance, indicating a significant change in the bacterial community structure in response to bacterial inoculation. PERMANOVA analysis confirmed that *Pseudomonas* sp. F204 inoculation significantly altered tomato rhizosphere bacterial community (PERMANOVA, *R*^2^ = 0.371, *P* = 0.009) (Fig. 3b).

### Effect of *Pseudomonas* sp. F204 Inoculation on the Tomato Rhizosphere Bacterial Community Composition

**Fig. 4:**
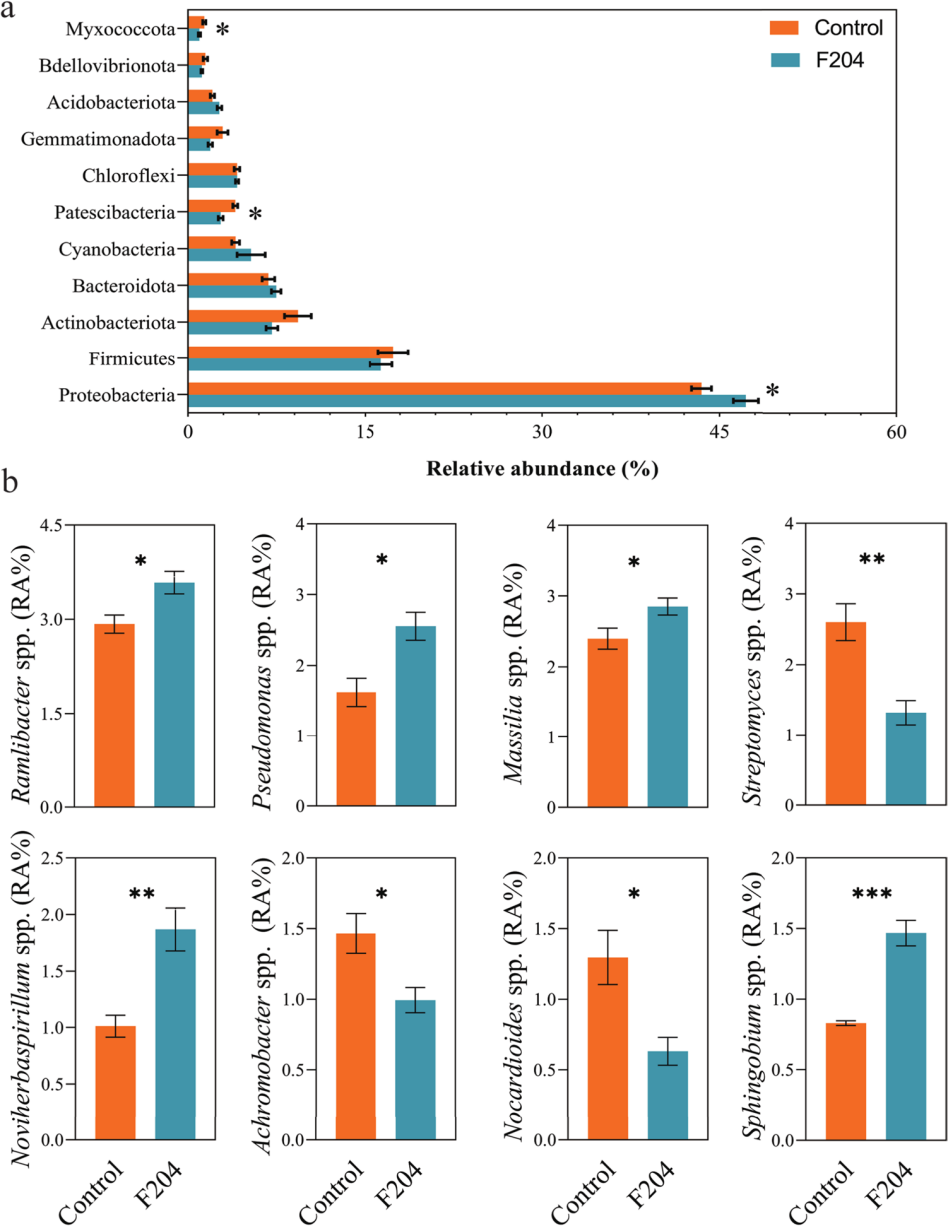
Relative abundances of major bacterial phyla (**a**) and altered bacterial genera (**b**) in tomato rhizosphere soils treated with *Pseudomonas* sp. F204 or control. Bacterial phyla with average relative abundance > 0.5% are shown and do not include unclassified taxa. Data are presented as the means of five independent replicates. The asterisk indicates statistically significant differences between treatments (Welch's *t*-test, *P < 0.05; **P < 0.01; ***P < 0.001)

At the phylum level, a total of 22 phyla were detected. The 11 most abundant phyla with a relative abundance higher than 1% were Proteobacteria, Firmicutes, Actinobacteriota, Bacteroidota, Cyanobacteria, Patescibacteria, Chloroflexi, Gemmatimonadota, Acidobacteriota, Bdellovibrionota, and Myxococcota, respectively (Fig. 4a). Compared to the control, inoculation of *Pseudomonas* sp. F204 increased the relative abundance of Proteobacteria while decreased that of Patescibacteria and Myxococcota (Welch's *t*-test, *P* < 0.05). At the genus level, the relative abundances of *Ramlibacter*, *Massilia*, *Pseudomonas*, *Noviherbaspirillum*, and *Sphingobium* were significantly higher in the treatment group, while those of *Streptomyces*, *Achromobacter*, and *Nocardioides* were decreased (Welch's *t*-test, *P* < 0.05, Fig. 4b).

**Fig. 5:**
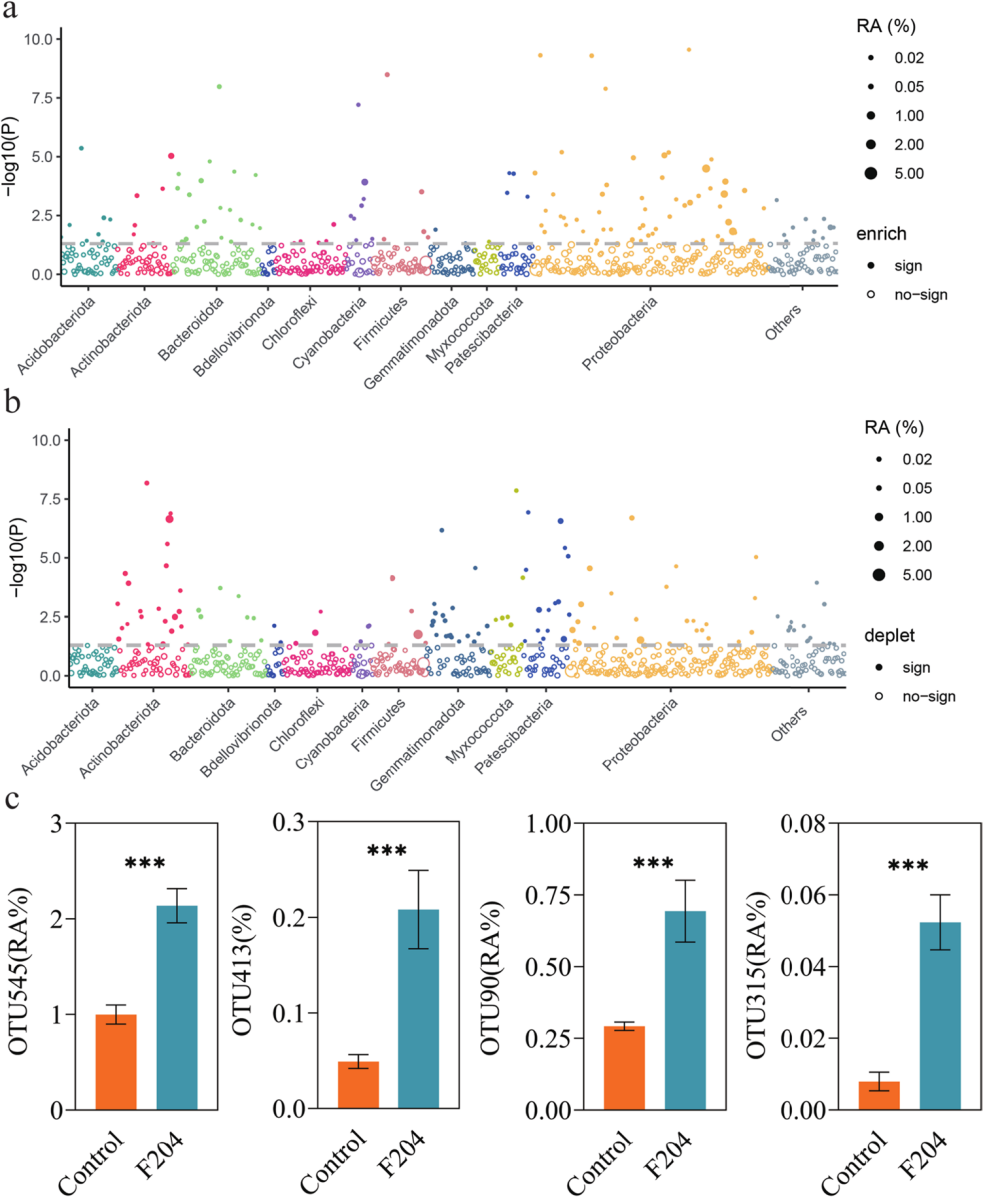
Differential OTUs in the tomato rhizosphere bacterial community and their classification at the phylum level. Manhattan plot showing the classification information of OTUs enriched (**a**) and depleted (**b**) in the rhizosphere of tomatoes treated with *Pseudomonas* sp. F204 compared to the control treatment. The dashed line represents the significance threshold (Welch's *t*-test, *P* = 0.05). **c** The relative abundance of most influenced OTUs by *Pseudomonas* sp. F204 treatment. (Welch's *t*-test, ***P < 0.001)

Compared to the untreated samples, the treatment of *Pseudomonas* sp. F204 increased the relative abundance of 133 OTUs, mostly from Proteobacteria, and decreased that of 127 OTUs, mostly from Actinobacteriota and Proteobacteria (Fig. 5a and b). The OTUs that were highly positively influenced by *Pseudomonas* sp. F204 treatment were OTU545 (*Pseudomonas* sp.), OTU90 (*Arthrobacter* sp.), OTU414 (*Bacillus* sp.), and OTU315 (*Pseudomonas* sp.) (Welch's *t*-test, *P* < 0.05; Fig. 5c). Interestingly, OTU315 showed 98.83% sequence similarity to F204. The sequences of F204 and OTU315 are provided in Appendix A.

## Discussion

During crop production, plant or dispersed plant materials such as leaves, fruits, pollen, seeds, and shed root cap cells can release DNA molecules [39], and most of them are fragmented by nucleases into small fragments before reaching the rhizosphere [40], and affects the growth and development of plants. Certain microorganisms closely associated with plant rhizospheres, such as *Bacillus* and *Pseudomonas,* exhibit eDNase activity, either through active secretion or passive release, to degrade surrounding eDNA [12, 41]. *Pseudomonas* spp. are vital plant-associated bacteria that inhabit various ecological niches and exert diverse effects on plants. While some pathogens, such as strains of *Pseudomonas syringae* that are responsible for disease in many higher plants [42], most *Pseudomonas* spp. strains act as plant growth promoters to help control fungal and bacterial plant diseases [15, 43, 44]. We screened bacteria capable of degrading eDNA from tomato eDNA-treated soil using TBO-DNA agar medium and identified it as *Pseudomonas* sp. F204. The eDNase activity of F204 can be detected on agar plates supplemented with specific indicators such as methylene blue, where the formation of clear zones around colonies indicates eDNA degradation [41, 45]. Similar observations of DNA degradation by *Pseudomonas* strains have been reported previously [20].

The influence of most of the PGPRs on plant growth is critical because they function as biofertilizers, promoting nutrient uptake by enhancing root colonization with beneficial microorganisms [46]. These beneficial microorganisms also assist hosts through hormone production and iron chelation, thus supporting plant growth [44]. IAA is a plant hormone that plays an important role in physiological processes such as cell division, elongation, and root development [47]. In our study, *Pseudomonas* sp. F204 exhibited the ability of IAA production, siderophore production, and phosphate solubilization. These bacterial characteristics are widely regarded to contribute to the improvement of plant growth, stress resistance, and increased yield [15, 47]. We further confirmed the effect of this strain on tomato seedling growth through pot experiments. In partial support of our hypothesis, the results showed a remarkable increase in the dry biomass of tomato seedlings treated with this strain compared to the control group, indicating a substantial positive effect of this strain on tomato growth. Similarly, *Pseudomonas* strains have shown growth-promoting effects on several plant species, such as tomato and wheat [29, 48].

The rhizosphere microbiota represents the most biologically active zone of the root–soil interface, sensitively reflecting changes in the soil ecosystem and serving as a key indicator of plant health and soil function [49]. We further investigated the impact of *Pseudomonas* sp. F204 on the tomato rhizosphere bacterial community. Although our results showed that *Pseudomonas* sp. F204 had no significant effect on soil bacterial alpha diversity; it significantly altered the bacterial community structure and composition, which supports our hypothesis. In particular, the Proteobacteria phylum showed a significant increase. Proteobacteria are extremely widely distributed in terrestrial soil microbial communities and a eutrophic bacteria with some members specialized in nitrogen fixation [50] and environmental adaptability [51].

The use of beneficial microbes not only directly affects the soil microbial environment but also potentially influences ecosystem stability and functionality by altering the structure and activity of soil microbial communities [52, 53]. *Pseudomonas* sp. F204 might have influenced the soil bacterial community structure by increasing the abundance of beneficial plant-associated taxa. Therefore, the increased tomato growth in *Pseudomonas* sp. F204 treatment could be a combined effect of an increase in mostly plant-beneficial taxa in the tomato rhizosphere, and the direct role of *Pseudomonas* sp. F204 in the degradation of eDNA and plant growth promotion. Moreover, the *Pseudomonas* sp. F204 treatment also increased the abundance of OTU315, suggesting a potential correlation with the addition of F204. Studies have shown that *Pseudomonas* are adaptable gram-negative opportunistic bacteria that can rapidly adapt to the environment and become dominant species [54]. This strongly explains the significant enrichment of the relative abundance of OTU315 after isolate F204 was added to the soil, which showed positive effects on plant growth and soil bacterial community composition.

## Conclusion

In this study, a *Pseudomonas* sp. F204, capable of degrading eDNA was isolated from soil treated with fragmented tomato eDNA. In vitro assays showed that *Pseudomonas* sp. F204 has the ability of IAA and siderophore production and phosphate solubilization. The inoculation of tomato soil with this strain altered the structure and composition of the rhizosphere bacterial community by increasing the relative abundance of certain taxa. These results suggest that eDNA-degrading bacteria *Pseudomonas* sp. F204 can promote host plant growth and alter its rhizosphere microbial community. Engineering such microbes as biostimulants could aid in combating the phenomenon of autotoxicity largely caused by continuous monocultures.

## Supplementary Information

Below is the link to the electronic supplementary material.

Supplementary file1 (PDF 80 KB)

## Data Availability

The Illumina sequence data underlying this article are available in GenBank under BioProject accession number PRJNA1138627. The 16S rRNA gene sequence of strain F204 was submitted to NCBI under the accession number PQ638548. Summarized data underlying this article are available in the article and in its online supplementary material. The raw data will be made available on request.

## Code Availability

Code will be made available on request.
