# Supplementary material for: Pseudomonas sp. F204 Promoted Tomato Growth and Altered Rhizosphere Bacteria Community
Source: Curr Microbiol. 2025 Jul 12;82(9):382. doi: 10.1007/s00284-025-04278-y (PMC12254067; doi:10.1007/s00284-025-04278-y)
Supplement: Supplementary file 1 — Supplementary file1 (PDF 80 KB) [file 284_2025_4278_MOESM1_ESM.pdf]

Pseudomonas sp. F204 promoted tomato growth and altered rhizosphere bacteria community

journal name: Current Microbiology

Jiawei Li[a], Yingjie A[a], Minghao Liu[b], Xin Li[a], Yilin Zhan[c], Muhammad Khashi u Rahman[a,d]*, Xingang Zhou[a]*

[a] Key Laboratory of Biology and Genetic Improvement of Horticultural Crops (Northeast Region), Ministry of Agriculture and Rural Affairs, Department of Horticulture, Northeast Agricultural University, Harbin 150030, China.

[b] School for the Engineering of Matter, Transport and Energy, Arizona State University, Tempe, Arizona 85287, United States.

[c] College of Agriculture, Northeast Agricultural University, Harbin 150030, China.

[d] Department of Microbiology and Genetics & Institute for Agribiotechnology Research (CIALE), University of Salamanca, Salamanca 37007, Spain.

*Correspondence: Muhammad Khashi u Rahman (khashiurahman@yahoo.com), Xingang Zhou (xgzhou@neau.edu.cn)

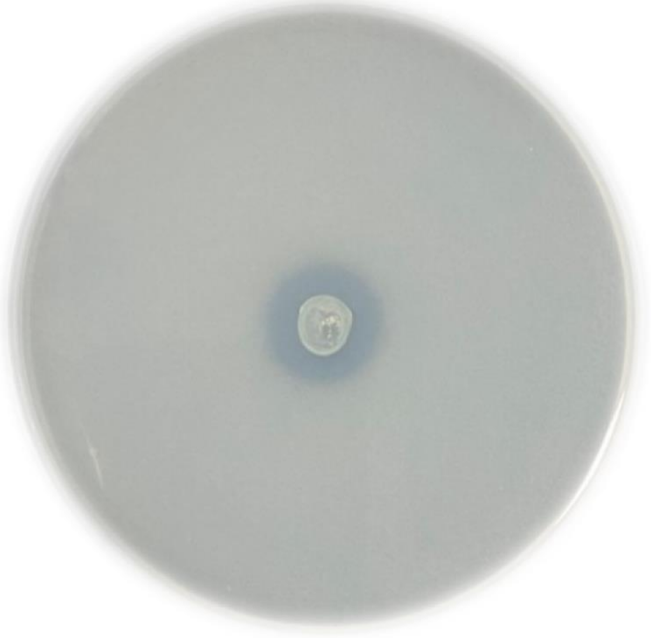

Fig. 1S Pseudomonas aeruginosa F204 phosphate solubilization test

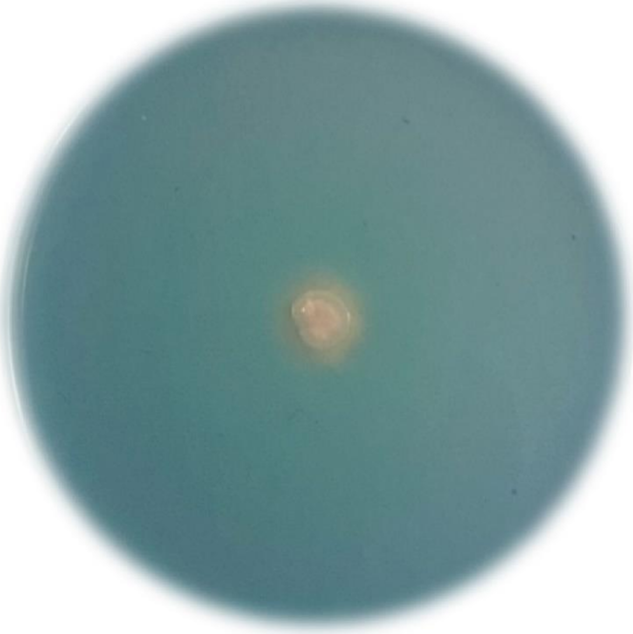

Fig. 2S Pseudomonas aeruginosa F204 Siderophore production test

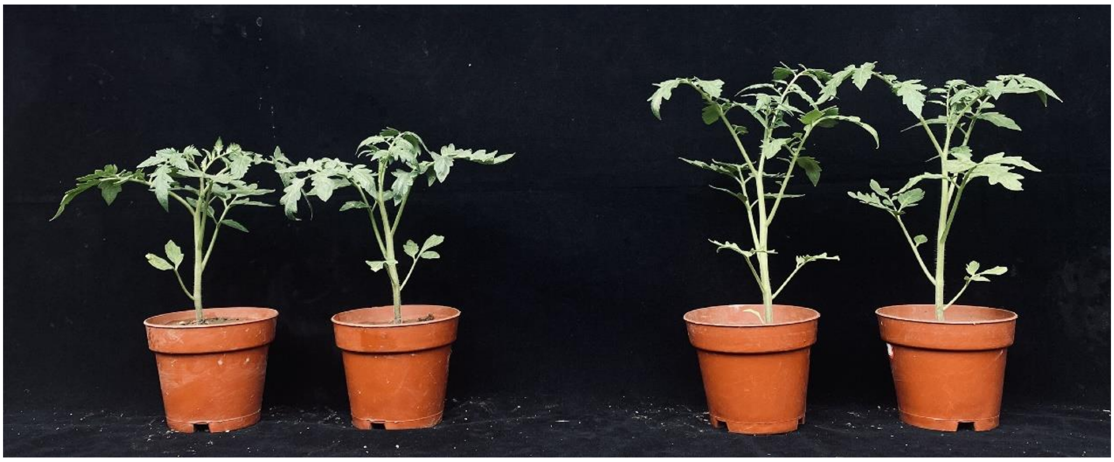

Fig. 3S Effect of Pseudomonas aeruginosa F204 on the growth of tomato seedlings. The two plants on the right are F204-treated

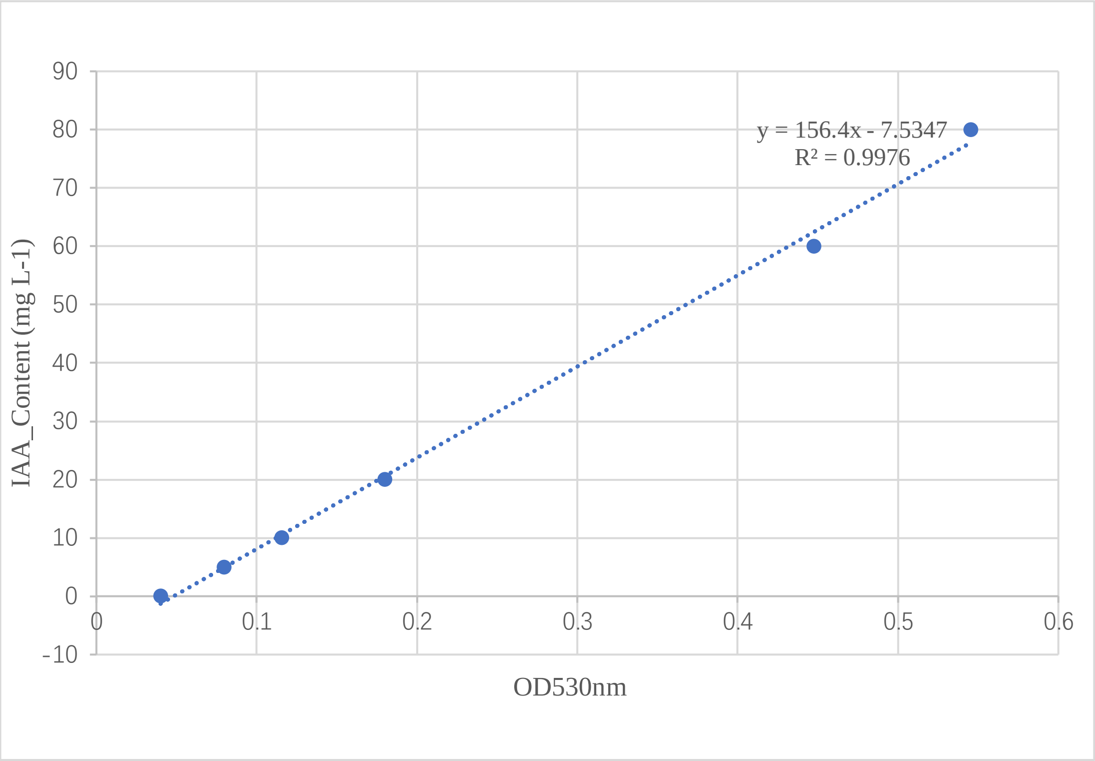

Fig. 4S IAA standard curve

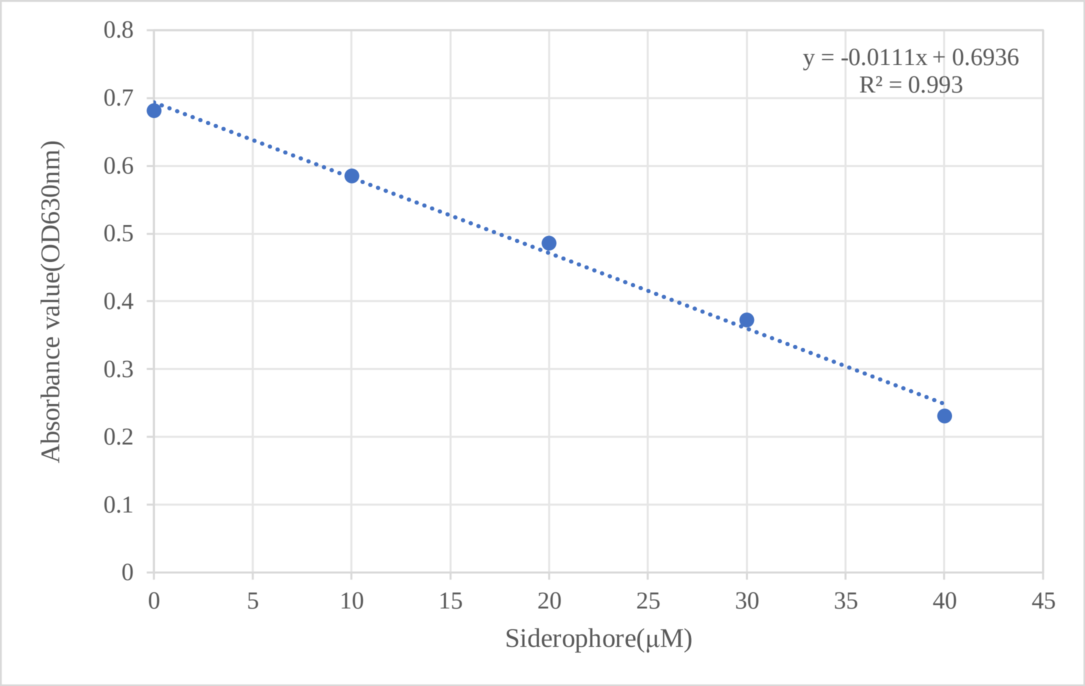

Fig. 5S Siderophore standard curve

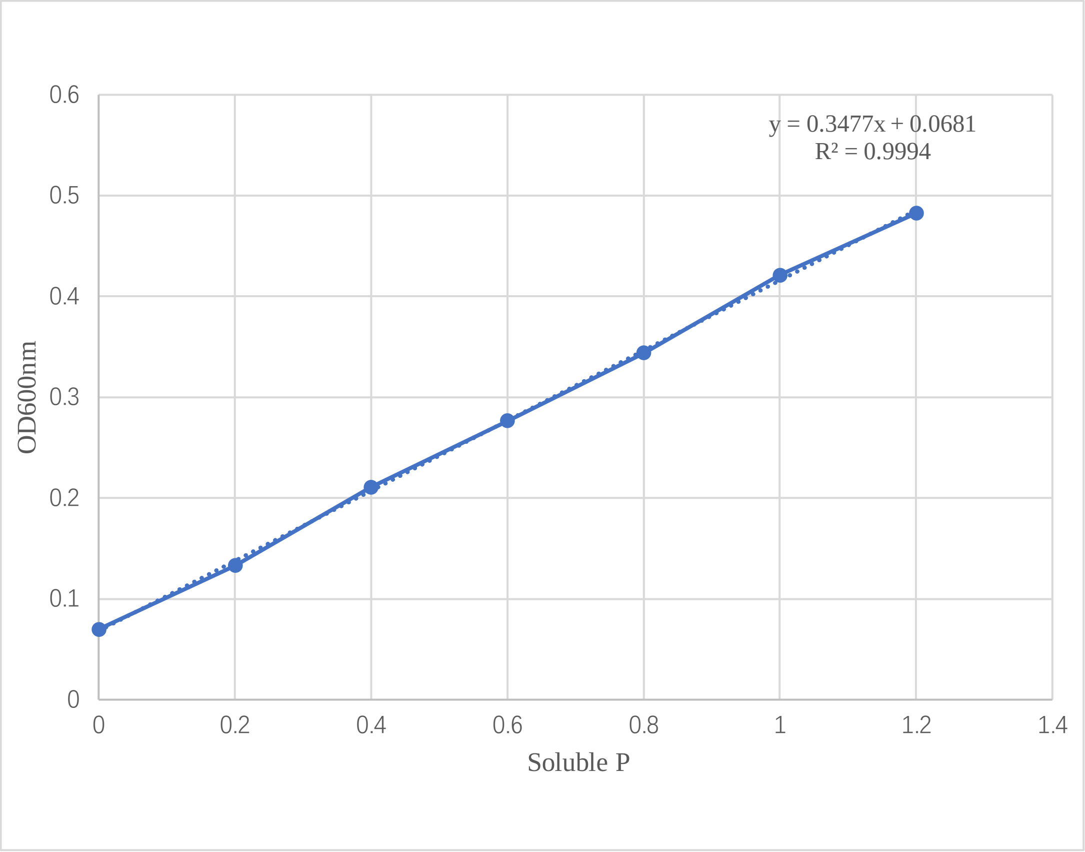

Fig. 6S Soluble P standard curve
